# Effects of 12 nutritional interventions on type 2 diabetes: a systematic review with network meta-analysis of randomized trials

**DOI:** 10.1186/s12986-025-00968-3

**Published:** 2025-08-07

**Authors:** Yi Liu, Haiyue Li, Qian Zhao, Wenxiang Cui

**Affiliations:** 1 School of Nursing, Yanbian University, Yanji City, Jilin Province, 133002 China; 2 Department of General Surgery, Yanbian University Hospital, Yanji City, Jilin Province, 133002 China

**Keywords:** Type 2 Diabetes, Nutritional Interventions, Network Meta-Analysis, Randomized Controlled Trials, Glycemic Control

## Abstract

**Background:**

Numerous trials confirm dietary interventions benefit type 2 diabetes mellitus (T2DM) management, but the optimal model is unclear. We evaluated 12 interventions through a Network Meta-Analysis (NMA) on their effects on Fasting Plasma Glucose (FPG), 2-h Postprandial Glucose (2hPG), HbA1c, Homeostasis Model Assessment of Insulin Resistance (HOMA-IR), Total Cholesterol (TC), Triglycerides (TG), and BMI, providing evidence to guide clinical nursing.

**Methods:**

We conducted an NMA of randomized controlled trials (RCTs) (PROSPERO registration: CRD42023429616), searching eight databases for studies published between January 1, 2010, and August 31, 2024. Two reviewers independently screened studies, extracted data, and assessed bias using the Cochrane Risk of Bias tool. Key and important outcomes were analyzed using Stata 17.0, with evidence quality assessed via the Grading of Recommendations Assessment, Development and Evaluation (GRADE) and Confidence in Network Meta-Analysis (CINeMA) scores.

**Results:**

Eighteen RCTs comprising 1,687 patients were included. Among 12 evaluated dietary interventions, MNT ranked highest in reducing FPG (SUCRA = 77.6%; SMD = -0.75; 95% CI: -0.88 to -0.61). Digital dietary models were most effective for reducing HbA1c (SUCRA = 84.6%; SMD = -1.06; 95% CI: -2.11 to -0.01), while LGI diets were superior for both 2hPG (SUCRA = 62.1%; SMD = -0.62; 95% CI: -0.76 to -0.47) and HOMA-IR (SUCRA = 96.9%; SMD = -10.13; 95% CI: -15.96 to -4.30). The LGI + LGL intervention was most effective in reducing TC (SUCRA = 88.3%), TG (SUCRA = 80.6%), and BMI (SUCRA = 99.8%), with statistically significant differences observed in pairwise comparisons (*P* < 0.05). The quality of evidence was rated as high for FPG, 2hPG, HbA1c, and BMI, and moderate for HOMA-IR, TC, and TG.

**Conclusions:**

These findings highlight the potential of MNT, LGI, digital dietary models, and LGI + LGL interventions to improve glycemic control and metabolic outcomes in patients with T2DM. However, further large-scale, multicenter RCTs are warranted to validate their long-term efficacy and safety.

**Trial registration:**

CRD42023429616.

**Supplementary Information:**

The online version contains supplementary material available at 10.1186/s12986-025-00968-3.

## Background

Type 2 diabetes mellitus (T2DM) is a chronic metabolic disorder characterized by insulin resistance and progressive β-cell dysfunction, leading to sustained hyperglycemia and increased risks of cardiovascular disease, nephropathy, neuropathy, and retinopathy [21]. The global burden of type 2 diabetes mellitus (T2DM) continues to grow, with projections estimating that over 783 million individuals will be affected by 2045 [48]. In China, the prevalence of diabetes among adults has reached 11.2%, with T2DM accounting for most of the cases [31]. Given the complexity of its pathogenesis and the long-term complications, lifestyle and dietary modifications remain foundational strategies in T2DM management [16].

Suboptimal dietary habits are important modifiable risk factors for type 2 diabetes mellitus (T2DM). Consequently, several dietary interventions have been developed to enhance glycemic control and metabolic health. Notably, both the Mediterranean and low-carbohydrate diets have proven effective in reducing fasting plasma glucose (FPG) and glycated hemoglobin (HbA1c) [56]. Low–glycemic‐index (LGI) diets, which emphasize slowly digestible carbohydrates, attenuate postprandial glucose excursions and enhance insulin sensitivity, as evidenced by reductions in the Homeostasis Model Assessment of Insulin Resistance (HOMA-IR) [13]. However, newer dietary models, such as digital interventions and the PCPA model (Phases: Advocacy, Building Alliances, Promotion and Mobilization, and Action), are emerging as potentially effective alternatives and require further evaluation.

Digital dietary models, including platforms such as Twin Precision Nutrition (TPN), use continuous glucose monitoring (CGM), food tracking and machine learning algorithms to provide personalized recommendations [53]. These approaches have been associated with significant improvements in HbA1c and patient adherence. Similarly, theory based frameworks such as the PCPA model promote long term dietary compliance through structured behavioral strategies and community based support [37]. Additionally, app based platforms like Foodsmart facilitate sustained dietary behavior change by offering real time feedback, individualized meal plans and food ordering options [54]. Thus, incorporating these less conventional but increasingly implemented strategies offers a broader and more current assessment of dietary interventions applicable to T2DM management in clinical settings.

Mechanistically, dietary interventions influence glycemic control and metabolic regulation through several interrelated pathways. LGI diets reduce postprandial glucose excursions by slowing carbohydrate digestion and absorption, which enhances insulin sensitivity [13]. They also lower HOMA-IR by modulating postprandial glycemic fluctuations and enhancing insulin signaling efficiency [66]. The inclusion of whole grains, legumes and non-starchy vegetables in LGI diets delays gastric emptying and reduces carbohydrate absorption rates, mitigating postprandial hyperglycemia and limiting insulin surges [57]. This more stable glycemic profile alleviates stress on pancreatic β cells and supports peripheral insulin sensitivity [63]. Additionally, LGI diets have been associated with reduced systemic inflammation and lower circulating free fatty acid levels, both of which interfere with insulin signaling pathways, further contributing to improved HOMA-IR [14].

Medical Nutrition Therapy (MNT) achieves glycemic benefits by optimizing macronutrient composition to reduce β-cell workload and enhance metabolic flexibility [52]. Digital dietary models offer real-time feedback based on individualized glycemic responses, allowing patients to dynamically adjust their dietary intake and reduce glucose variability [45]. The combined LGI and low glycemic load (LGL) dietary approach further improves metabolic outcomes by incorporating both glycemic index (GI) and load considerations, thereby minimizing lipid accumulation and lowering cardiovascular risk markers [61]. Although many randomized controlled trials (RCTs) have demonstrated the effectiveness of individual dietary strategies, existing meta-analyses primarily employ pairwise comparisons, which are limited in evaluating multiple interventions simultaneously [52]. Additionally, many prior reviews have excluded newer digital and model-based interventions. Thus, comprehensive network meta-analysis (NMA) that includes both conventional and emerging dietary strategies is needed to provide a clearer evidence-based ranking of effectiveness across a range of outcomes.

This study hypothesizes that dietary interventions differ in their effectiveness in improving glycemic and metabolic outcomes among patients with type 2 diabetes mellitus. The aim of this network meta analysis is to systematically evaluate and compare the efficacy of 12 dietary interventions including both traditional and emerging strategies on key clinical outcomes such as fasting plasma glucose (FPG), 2 h postprandial glucose (2hPG), glycated hemoglobin (HbA1c), Homeostasis Model Assessment of Insulin Resistance (HOMA IR), total cholesterol (TC), triglycerides (TG) and body mass index (BMI). By synthesizing direct and indirect evidence this analysis will generate a ranked comparison to guide dietary recommendations in clinical practice.

## Methods

### Registration

This study was prospectively registered in the PROSPERO International Systematic Review Database (https://www.crd.york.ac.uk/prospero/, accessed on August 31, 2024) under the registration number CRD42023429616. The study’s design, execution, and reporting adhered to the Preferred Reporting Items for Systematic Reviews and Meta-Analyses (PRISMA) guidelines, as well as the specific recommendations NMA [3, 24].

### Search strategy

A comprehensive literature search was conducted across both Chinese and English databases. Chinese databases included CNKI, WanFang, VIP, and SinoMed, while English-language databases included Web of Science, PubMed, Medline, and the Cochrane Central Register of Controlled Trials (CENTRAL). The search covered publications from January 1, 2010, to August 31, 2024. We selected this period to emphasize contemporary evidence reflecting current clinical practice, recent advances in nutritional science and digital interventions, and to adequately capture the evolution of dietary approaches for managing T2DM over the past decade.

The search strategy incorporated both Medical Subject Headings (MeSH) and free-text terms, combining them using Boolean operators (AND, OR) to improve sensitivity and specificity. Terms related to “nutritional intervention,” “nutrition policy,” and “type 2 diabetes mellitus (T2DM)” were used in conjunction with trial-related keywords such as “randomized controlled trial” and “RCT.” Separate search strategies were tailored for each database. A summary of the core search terms in Chinese and English databases is presented in Table 1, and the detailed PubMed search string is provided in Supplementary Table 1.

**Table 1:** General information about the included studies

No	Included Study	Country/Region	Sample Size (cases) T/C	T1 Intervention	T2 Intervention	Control Intervention	Outcome Indicators
1	Yang Bing, 2021 [65]	China (Shaanxi)	T = 47/C = 48	Low-carbohydrate dietary intervention (LCD)	–	Low-fat dietary intervention (LFD)	➀➁➂➄➅
2	Sang-Man Jin, 2021 [26]	South Korea	T1 = 16/T2 = 17/C = 20	East Asia alternative diet (EAAD)	Korean Food Exchange Mode (KFEM)	Routine diabetes dietary intervention (UC)	➂
3	Yuka Omura, 2021 [42]	Japan	T = 64/C = 62	Personalized medical nutrition therapy (MNT)	–	Routine diabetes dietary intervention (UC)	➂
4	Chen Xianghua, 2020 [4]	China (Guangdong)	T = 40/C = 40	Carbohydrate counting (CHO)	–	Routine diabetes dietary intervention (UC)	➀➂➄➅
5	Li Xiaotao, 2020 [30]	China (Ningxia)	T = 63/C = 48	Low-carbohydrate dietary intervention (LCD)	–	Routine diabetes dietary intervention (UC)	➀➁➂➆
6	Xu [64]	China (Shanghai)	T = 48/C = 48	Digital nutrition dietary intervention (DN)	–	Routine diabetes dietary intervention (UC)	➀➁➂
7	Nivedita Pavithran, 2020 [44]	India	T = 18/C = 18	Low-glycemic index dietary intervention (LGI)	–	Routine diabetes dietary intervention (UC)	➂➄➅
8	Ma [49]	China (Shijiazhuang)	T = 62/C = 60	Personalized medical nutrition therapy (MNT)	–	Routine diabetes dietary intervention (UC)	➀➁➂➃➄➅➆
9	Rosario, 2019 [2]	Spain	T = 94/C = 91	Multi-factor + Mediterranean diet (EMID)	–	Routine diabetes dietary intervention (UC)	➂
10	Wang Ruiping, 2015 [50]	China (Kunming)	T1 = 27/T2 = 29/C = 29	Low-glycemic index dietary intervention (LGI)	Water-soluble dietary fiber intervention (WSDF)	Routine diabetes dietary intervention (UC)	➀➁➂➃➄➅
11	Wang Xia, 2015 [59]	China (Nanjing)	T = 50/C = 50	Low-glycemic index & low-glycemic load dietary intervention (LGI + LGL)	–	Routine diabetes dietary intervention (UC)	➀➁➂➄➅➆
12	Chang Na, 2014 [39]	China (Jilin)	T = 36/C = 39	Personalized medical nutrition therapy (MNT)	–	Routine diabetes dietary intervention (UC)	➁➂➆
13	Quan Xiaojuan, 2014 [62]	China (Shaanxi)	T = 50/C = 50	Personalized medical nutrition therapy (MNT)	–	Routine diabetes dietary intervention (UC)	➀➂➄➅
14	Yao Li, 2013 [32]	China (Ningxia)	T = 48/C = 47	PCPA dietary intervention (PCPA)	–	Routine diabetes dietary intervention (UC)	➄➅
15	Ma Li, 2012 [35]	China (Ningxia)	T = 48/C = 47	PCPA dietary intervention (PCPA)	–	Routine diabetes dietary intervention (UC)	➀➆
16	Chen Min, 2012 [34]	China (Shanghai)	T = 62/C = 79	Low-glycemic index dietary intervention (LGI)	–	Routine diabetes dietary intervention (UC)	➀➁➂➄
17	Han Mingming, 2011 [38]	China (Tianjin)	T = 9/C = 9	Personalized medical nutrition therapy (MNT)	–	Routine diabetes dietary intervention (UC)	➀➁
18	Xu Danfeng, 2010 [10]	China (Shanghai)	T = 62/C = 61	Personalized medical nutrition therapy (MNT)	–	Routine diabetes dietary intervention (UC)	➀➁➂➃➄➅

Outcome Indicators: ➀ FPG (fasting plasma glucose) ➁ 2hPG (2-h postprandial glucose) ➂ HbAlc (glycosylated hemoglobin) ➃ HOMA-IR (homeostasis model assessment-insulin resistance) ➄ TC (total cholesterol) ➅ TG (triglycerides) ➆ BMI (body mass index)

*Abbreviations*: *UC* Routine diabetes dietary intervention, *LCD* Low-carbohydrate dietary intervention, *LFD* Low-fat dietary intervention, *EAAD* East Asia alternative diet, *KFEM* Korean Food Exchange Mode, *MNT* Personalized medical nutrition therapy, *CHO* Carbohydrate counting, *DN* Digital nutrition dietary intervention, *LGI* Low-glycemic index dietary intervention, *EMID* Multi-factor + Mediterranean diet intervention, *WSDF* Water-soluble dietary fiber intervention, *LGI* + *LGL* Low-glycemic index & low-glycemic load dietary intervention, *PCPA* PCPA dietary intervention

#### Inclusion crieria

P: T2DM; no restrictions on gender, nationality, or ethnicity.

I: 12 types of dietary interventions:

- Low Carbohydrate Diet Intervention [11]: total daily caloric intake was controlled at 1980 kcal, with carbohydrates, fats and proteins accounting for 33%, 45%, and 22% of total energy intake, respectively.
- Low Fat Diet Intervention [55]: Total daily caloric intake controlled at 1860 kcal, with carbohydrates, fats, and proteins accounting for 48%, 32%, and 20%, respectively.
- East Asian Alternative Diet Model [27]: sugar and starch intake were restricted, with an energy ratio of carbohydrates : fats : proteins of 4 : 3 : 3, and net carbohydrates contributing 27 percent of total energy intake.
- Korean Food Exchange Model [33]: energy ratio of carbohydrates : fats : proteins was 6 : 2 : 2, with sodium intake limited to 600–800 mg per meal.
- MNT [36]: individualized dietary guidance based on glycemic control, lipid profile, body weight and physical activity, with an emphasis on increasing protein intake.
- Carbohydrate Counting Method [28]: Carbohydrates, proteins, and fats comprised 55%, 20%, and 25% of the total caloric intake, respectively, distributed as 1/5, 2/5, and 2/5 across three meals.
- Digital Dietary Model [60]: Digital dietary interventions use AI and IoT based platforms to deliver personalized nutrition guidance via continuous glucose monitoring (CGM), food photo recognition and intelligent meal planning [37, 53] These systems generate individualized recommendations in real time by accounting for patients’ glycemic responses, dietary habits and behavioral inputs. Evidence suggests that sustained digital interventions lasting longer than six months not only reduce HbA1c but also enhance patient adherence. Ongoing algorithmic optimization supports long term glycemic stability (Merino Barbancho, 2022; [53, 54]).
- LGI Dietary Intervention [66]: A diet with a GI ≤ 45 that fully replaces breakfast and dinner.
- Multifactorial Mediterranean Diet Intervention [22]: Recommends white meat, four or more tablespoons of olive oil per day (1 tablespoon = 13.5 grams), two or more servings of vegetables, three or more servings of fruits, one or fewer servings of red meat or sausage, less animal fat, and less than one cup (100 mL) of carbonated or sugary drinks.
- Soluble Dietary Fiber Intervention [1]: Add 10 g of soluble dietary fiber to breakfast and dinner.
- LGI+LGL Dietary Intervention [17]: Total daily GI = Σ(food GI × intake amount × available carbohydrate %) ÷ total available carbohydrate amount of all foods; Total daily Glycemic Load (GL) = Σ(food GI × intake amount × available carbohydrate %) ÷ 100. The average GI and GL of 3-day dietary intake are used.
- PCPA dietary intervention [47]: This framework comprises four sequential phases—advocacy, coalition building, health promotion and action implementation [43]. The intervention includes community-based health education, formation of peer support groups and promotion of dietary change through repeated counselling and public awareness campaigns. In a 12-month community trial involving individuals with T2DM, this model produced significant improvements: a mean reduction in fasting plasma glucose of 1.2 mmol/L, a mean HbA1c decrease of 0.9 % and a 30 % increase in diabetes-related nutritional knowledge scores.

C: Conventional diabetes dietary intervention.

O: (a) Blood Glucose Control Indicators: FPG; Postprandial 2-h Glucose (2hPG); HbA1c; Insulin Resistance Index (HOMA-IR)

(b) Cardiovascular Risk Factors Indicators: TC; TG; BMI

S: RCT

The classification of the 12 dietary interventions was predefined prior to analysis and based on both nutritional content and delivery modality, in alignment with national and international dietary guidelines for T2DM (e.g., ADA 2022 [8]; Chinese Nutrition Society 2022 [6]; Hou et al. 2023 [19]; Iizuka and Yabe 2023 [25]; Nolan et al. 2022 [41]; Chinese et al. 2024 [5]). Interventions were categorized into macronutrient based diets (e.g., low carbohydrate, low fat), behavioral models (e.g., PCPA), GI based approaches (e.g., LGI, LGI + LGL) and technology assisted formats (e.g., digital dietary model). Specific caloric thresholds (e.g., 1860 or 1980 kcal per day) were derived from the original trial protocols to ensure fidelity to the reported interventions. The LGI intervention, which focused on replacing breakfast and dinner, reflects the structure used in the source studies where these meals contributed the majority of glycemic load. These categorizations were applied consistently across studies and were not determined based on post hoc performance, thereby preserving the integrity of the NMA framework.

#### Exclusion criteria

1. The study design is a cohort study, cross-sectional study, or case–control study;
2. Although the study is a clinical control trial, the grouping lacks randomization, or it is a non-synchronous clinical control study or a self-before-and-after control study;
3. Studies were excluded if they enrolled participants with type 1 diabetes, other specific diabetes subtypes, gestational diabetes or high-risk populations for diabetes. In this review, “high-risk populations” refers to individuals with prediabetes or those exhibiting one or more major risk factors for diabetes onset, including age ≥ 40 years, a family history of diabetes, overweight or obesity (especially central adiposity), hypertension, dyslipidemia, a sedentary lifestyle, a history of delivering a macrosomic infant or prior gestational diabetes. These groups were excluded to minimize heterogeneity arising from divergent disease progression rates and variable responses to dietary interventions, thereby ensuring comparability of effects among patients with established type 2 diabetes mellitus.
4. Study that lacked relevant outcome indicators;
5. Studies were excluded if they were reviews, commentaries, editorials, case reports, secondary analyses of primary research, or non-human trials.

### Data extraction

Two researchers (L and L) followed the search strategy to screen titles and assess relevant studies. Any unclear or ambiguous data in the original texts prompted full-text review. In cases of disagreement, a third researcher (C) conducted in-depth analysis, ensuring objective, accurate final assessments. Microsoft Excel was used to record the first author’s name, country/region, sample size, intervention/control measures, and outcome indicators.

### Risk of bias assessment

The Cochrane risk of bias tool was used to assess bias across seven domains, categorizing the risk as high, low, or unclear. After completing the assessments, Kappa consistency tests were conducted. Two researchers (L and L) independently evaluated the risk of bias for the final selected studies. Given the inherent difficulties in blinding in diet pattern RCTs, any differences were resolved by discussing with a third team member (C) until consensus was reached.

### Dealing with missing data

When standard deviation values were not directly reported, we estimated them from available statistical data in accordance with the Cochrane Handbook guidelines [20]. Specifically, when standard errors (SE) were reported, SDs were calculated using the formula $SD=SE\times\sqrt{n}$, where *n* represents the sample size. If *p*-values were provided without explicit SDs or SEs, we derived the corresponding t-statistic or z-score and applied the appropriate formulae to back-calculate SD values: $s=\frac{t\cdot\sqrt{n}\cdot(\mu-X)}{1}$, where *t* is the t-statistic derived from the reported *p*-value and degrees of freedom, and $\mu-X$ represents the difference in means. We estimated missing standard deviations to include as many studies as possible in the meta-analysis. Whenever data allowed, we verified those estimates against confidence intervals and test statistics to ensure accuracy.

### Statistical analysis

Traditional meta-analysis and NMA were conducted using Stata 17.0 software to evaluate the effects of 12 dietary interventions on clinical outcomes in patients with T2DM, including FPG, 2hPG, HbA1c, HOMA-IR, TC, TG, and BMI. Effect sizes were reported as mean difference (MD), standardized mean difference (SMD), odds ratio (OR), or risk ratio (RR), with corresponding 95% confidence intervals (CI).

Heterogeneity was assessed using the Cochran Q test and I^2^ statistic, with I^2^ > 50% indicating significant heterogeneity; in such cases, a random-effects model was applied [29], whereas a fixed-effects model was used otherwise. To further investigate the sources of heterogeneity, meta-regression analyses were conducted using a random-effects meta-regression model. The covariates included publication year, intervention duration, sample size, and risk of bias score. The reduction in heterogeneity was evaluated based on changes in τ^2^ and I^2^ values, and meta-regression results provided a statistical basis for identifying variables contributing to variation in effect sizes.

Node sizes in the network diagram corresponded to intervention sample sizes, while line thickness reflected the number of studies comparing each pair of interventions. Local inconsistency between direct and indirect evidence was assessed via node splitting, with comparisons exhibiting P values above 0.05 deemed consistent and analyzed under a consistency model. Probabilities of each intervention being most effective were quantified by the surface under the cumulative ranking curve (SUCRA) [7], where values range from 0 to 1 and higher values denote greater efficacy. These SUCRA-derived rankings were subsequently used to compare the relative performance of the 12 dietary interventions across the seven clinical outcomes.

### Sensitivity analyses

Given that NMA concerns closed loops, we tested for inconsistency between direct and indirect evidence using statistical methods specific to closed loops to detect potential discrepancies [23]. The stability of the combined results using both random-effects and fixed-effects models was assessed. Inconsistencies between these models suggested potential instability in the original findings. Sensitivity analyses were conducted by sequentially excluding individual studies to monitor changes in the combined results, thereby evaluating the influence of specific studies on the overall outcome. Furthermore, we examined the impact of low-quality studies by excluding them from the analysis.

### Credibility of the evidence

Funnel plots were created to analyze publication bias. The Grading of Recommendations Assessment, Development and Evaluation (GRADE) system was used to rate the quality of evidence for traditional meta-analysis results, and the Confidence in Network Meta-Analysis (CINeMA) online tool was used to assess the quality of evidence for NMA [46]. The combined effects for key outcomes (FPG, 2hPG, HbA1c) and important outcomes (HOMA-IR, TC, TG, BMI) were analyzed. CINeMA provided six ratings: within-study bias, reporting bias, indirectness, imprecision, heterogeneity, and incoherence [40].

## Results

### Search results and study selection

**Fig. 1:**
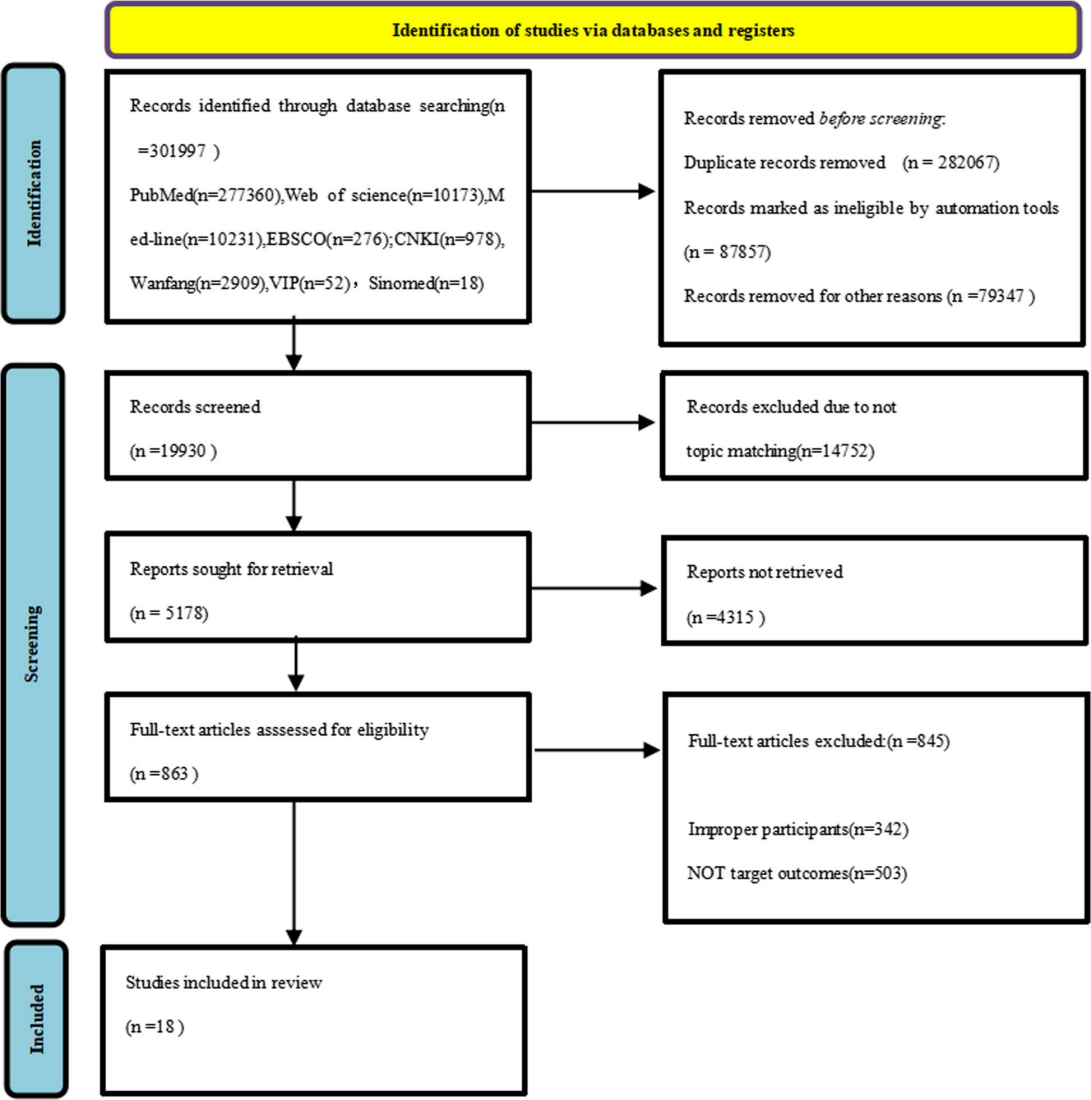
Flow diagram of the study selection

As of August 31, 2024, a total of 301,997 relevant articles were identified (3,957 in Chinese, 298,040 in English). After excluding duplicate publications and those that did not meet the inclusion criteria (19,930 articles), 3,135 articles were retained for further evaluation. Following a review of titles and abstracts, 18 RCTs were included [2, 4, 10, 26, 30, 32, 34, 35, 38, 39, 42, 44, 49, 50, 59, 62, 64, 65], consisting of 14 Chinese studies and 4 English studies. These studies involved 12 different dietary nutritional interventions. The literature screening flowchart is shown in Fig. 1,Table 1.

### Study characteristics

A total of 18 studies were included, with 4 studies conducted in South Korea, Japan, India, and Spain, and 14 studies conducted in China. The participants were primarily overweight and obese with T2DM. The studies were conducted by teams of clinical healthcare professionals or professional nutritionists. Sixteen of the studies were two-arm trials, while two were three-arm trials. The outcome indicators included: FPG, 2hPG, HbA1c, HOMA-IR, TC, TG, and BMI. The basic characteristics of the included studies are shown in Table 1.

### Risk of bias in included studies

The quality assessment was conducted using Cochrane 5.4.0 guidelines, with an inter-rater consistency Kappa value of 0.897. Eleven studies [2, 10, 26, 34, 38, 39, 42, 44, 49, 50, 62, 64] reported the random sequence generation method, while the others only mentioned randomization in the abstract. Seven studies [10, 32, 38, 42, 44, 49, 62] provided information on sample dropout rates and reasons, and the data were mostly complete. The included studies reported both primary and secondary outcome indicators in full, as summarized in Table 2.

**Table 2:** Included Study Quality Evaluation (Cochrane Handbook5.3.0)

No	Included Study	Selection Bias		Performance Bias	Detection Bias	Attrition Bias	Reporting Bias	Other Bias	Jadad Score
1	Yang Bing, 2021 [65]	3	3	3	3	1	1	1	4
2	Sang-Man Jin, 2021 [26]	1	2	1	1	1	1	1	6
3	Yuka Omura, 2021 [42]	1	1	1	1	1	1	1	6
4	Chen Xianghua, 2020 [4]	2	3	3	3	1	1	1	4
5	Li Xiaotao, 2020 [30]	3	3	3	3	1	1	1	4
6	Xu [64]	1	2	1	1	1	1	1	5
7	Nivedita Pavithran, 2020 [44]	1	1	1	1	1	1	1	6
8	Ma [49]	1	3	3	3	1	1	1	4
9	Rosario, 2019 [2]	1	2	1	1	1	1	1	6
10	Wang Ruiping, 2015 [50]	1	2	2	2	1	1	1	4
11	Wang Xia, 2015 [59]	1	2	1	1	1	1	1	7
12	Chang Na, 2014 [39]	1	1	1	1	1	1	1	7
13	Quan Xiaojuan, 2014 [62]	1	1	1	1	1	1	1	7
14	Yao Li, 2013 [32]	3	3	3	3	1	1	1	4
15	Ma Li, 2012 [35]	1	3	3	3	1	1	1	4
16	Chen Min, 2012 [34]	1	1	1	1	1	1	1	7
17	Han Mingming, 2011 [38]	1	1	1	1	1	1	1	7
18	Xu Danfeng, 2010 [10]	1	1	1	1	1	1	1	7

1 = Low risk; 2 = High risk; 3 = Unclear

### Traditional meta-analysis results

Using Stata 17.0, a traditional meta-analysis was performed for outcome indicators supported by at least two direct comparison studies [68]. All 12 nutritional interventions demonstrated statistically significant effects (*P* < 0.05) compared with conventional diabetes dietary interventions. Given the presence of substantial heterogeneity, a random -effects model was employed for all analyses.

Potential sources of heterogeneity were identified, including variability in measurement tools, differences in intervention duration, and heterogeneity in dietary implementation protocols. Methodological factors such as lack of strict randomization, allocation concealment, and blinding procedures were also recognized as contributing factors (see Supplementary Materials).

To further illustrate the degree of heterogeneity, I^2^ values for the primary outcomes were as follows: FPG (I^2^ = 66.4%), 2hPG (I^2^ = 52.7%), HbA1c (I^2^ = 58.1%), HOMA-IR (I^2^ = 49.6%), TC (I^2^ = 60.3%), TG (I^2^ = 55.2%), and BMI (I^2^ = 50.4%). Meta-regression analyses indicated that intervention duration (*p* = 0.017) and study quality (*p* = 0.041) were statistically significant moderators of heterogeneity. Full heterogeneity statistics and meta-regression outputs are provided in Supplementary Table 1, 2.

### Effects of the interventions

The NMA results were reported based on the CINeMA evidence grading framework [58]. In the constructed network diagrams, each node represents a dietary intervention, with node size and line thickness corresponding to the cumulative sample size and number of studies for each comparison, respectively. Direct comparisons were indicated by solid lines, while indirect comparisons were inferred based on the network structure. Node color reflected the overall risk of bias: red for high, yellow for moderate, and green for low, in accordance with Cochrane risk-of-bias assessments.

For each of the seven outcomes—➀FPG, ➁ 2hPG, ➂ HbA1c, ➃ HOMA-IR, ➄ TC, ➅ TG, and ➆ BMI—the overall inconsistency test indicated *P* > 0.05, and the node-splitting method confirmed no significant inconsistency across comparisons. Specifically, node-splitting results for key comparisons showed consistent findings between direct and indirect evidence. For example, the comparison between LGI and MNT for HOMA-IR yielded *P* = 0.289; the digital dietary model versus conventional diet for HbA1c had *P* = 0.371; and LGI + LGL versus low-carbohydrate diets for BMI resulted in *P* = 0.456. All comparisons had P-values > 0.05, confirming that the consistency assumption held throughout the network. Full node-splitting results for all outcomes are detailed in Supplementary Table 1, 2.

**Fig. 2:**
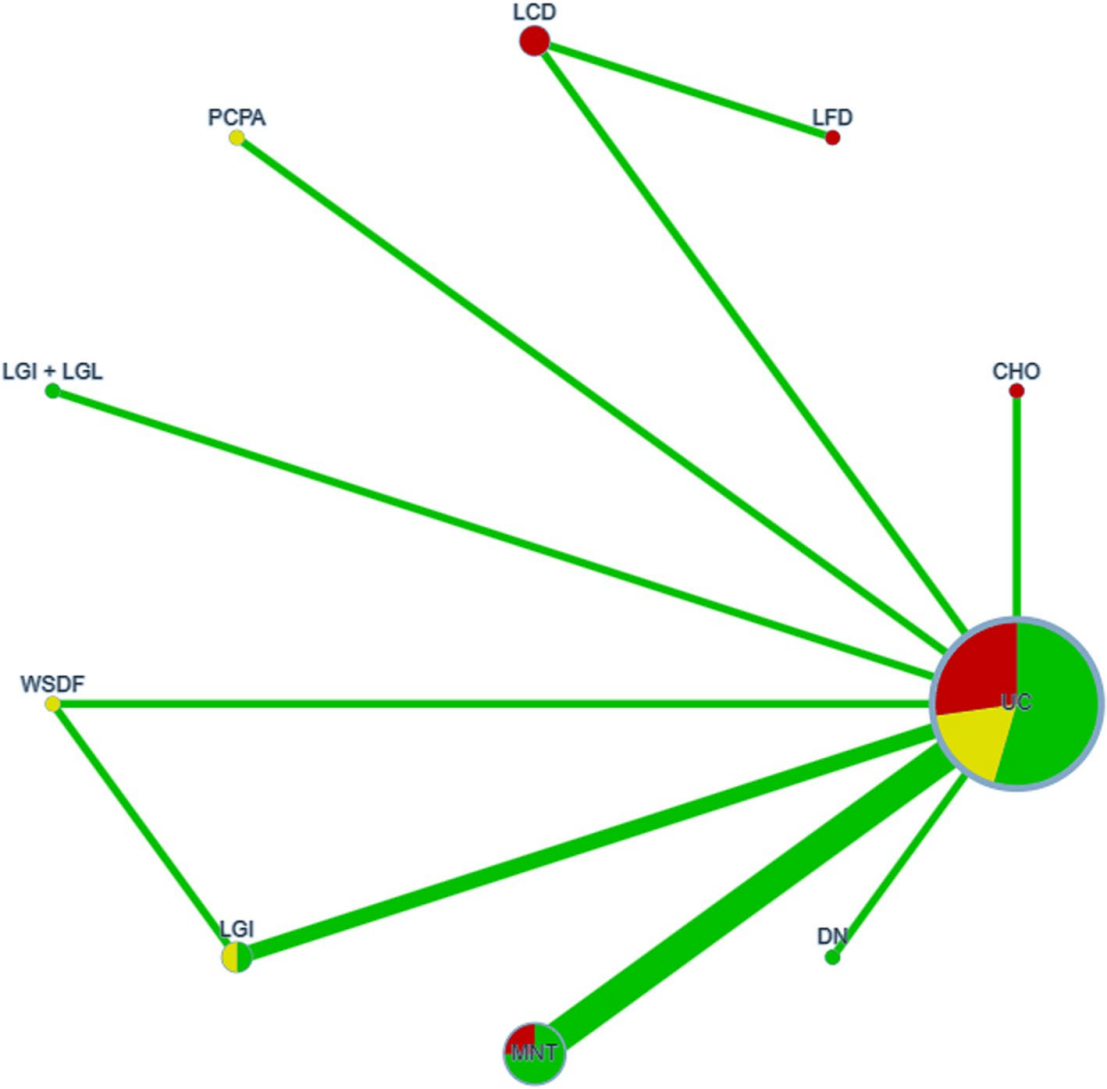
Network meta-analysis evidence network diagram for FPG

**Fig. 3:**
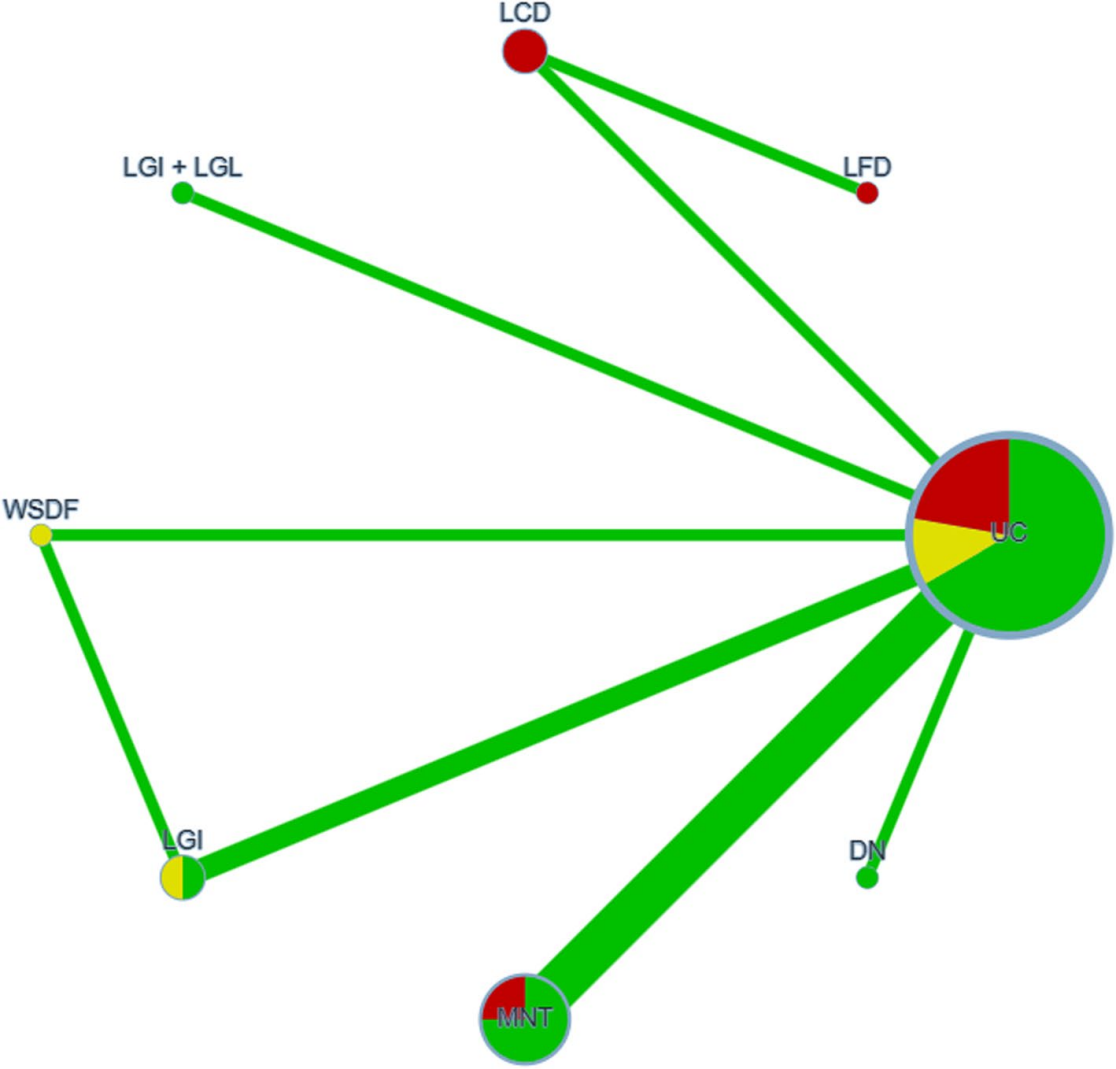
Network meta-analysis evidence network diagram for 2hPG

The consistency model was thus adopted for the final analysis. Based on SUCRA values, the highest-ranking interventions were as follows: MNT for reducing FPG (77.6%), digital dietary models for HbA1c (84.6%), LGI diets for both 2hPG (62.1%) and HOMA-IR (96.9%), and LGI + LGL diets for TC (88.3%), TG (80.6%), and BMI (99.8%) (all *P* < 0.05). The detailed SUCRA rankings are presented in Table 3 and Figs. 2, 3.

**Table 3:** Effects of the Interventions

	The SUCRA ranking	The inconsistency test (> 0.05)	node-splitting method	Model
FPG[17, 20-22, 24, 26, 27, 29, 31, 32, 34]	➅(77.6%) > ⑬(61.3%) > ➁(59.4%) > ➇(58.4%) > ➆(55.6%) > ➂(54.6%) > ⑫(53.3%) > ➈(43.8%) > ➀(19.9%) > ⑪(16.1%)	*P* = 0.142	*P* > 0.05	The consistency model
2hPG[17, 21, 22, 24, 26–28, 32–34]	➈(62.1%) > ➅(61.0%) > ⑪(52.1%) > ➂ (50.7%) > ⑫(49.5%) > ➇ (49.2%) > ➁(49%) > ➀(26.4%)	*P* = 0.648	*P* > 0.05	The consistency model
HbA1c[17–29, 32, 34]	➇(84.6%) > ➅ (75.4%) > ⑫(72.1%) > ➆ (71.1%) > ➈(65.3%) > ⑪(52.4%) > ➂(41.2%) > ➁(39.5%) = ➉(39.5%) > ➀(35.9%) > ➄(17.1%) > East Asian ➃(6%)	*P* = 0.983	*P* > 0.05	The consistency model
HOMA-IR[24, 26, 34]	➈(96.9%) > ⑪(64.4%) > ➅ (32.8%) > ➀(5.9%)	*P* = 0.165	*P* > 0.05	The consistency model
TC[17, 20, 23, 24, 26, 27, 29, 30, 32, 34]	⑫(88.3%) > ➅(70.1%) > ⑬ (46.3%) > ➆(45.1%) > ➈(45.0%) > ➀ (34.0%) > ⑪ (21.3%)	*P* = 0.188	(*P* > 0.05)	The consistency model
TG[17, 20, 23, 24, 26, 27, 29, 30, 32, 34]	⑫(80.6%) > ➈ (71.0%) > ➅ (57.8%) > ⑪(45.4%) > ⑬(26.5%) > ➀ (6.4%)	*P* = 0.341	(*P* > 0.05)	The consistency model
BMI[21, 24, 27, 28, 31]	⑫(99.8%) > ➁(62.6%) > ➅(42.1%) > ⑬(28.0%) > ➀ (17.5%)	*P* = 0.923	(*P* > 0.05)	The consistency model

➀ conventional diabetes diet ➁ low-carb diet ➂ low-fat diet ➃ East Asian alternative diet ➄ Korean food exchange model ➅ MNT ➆ carbohydrate counting ➇ digital dietary patterns ➈ LGI diet ➉ multi-factor Mediterranean diet intervention ⑪ water-soluble dietary fiber intervention ⑫ LGI + LGL ⑬ PCPA dietary intervention

**Fig. 4:**
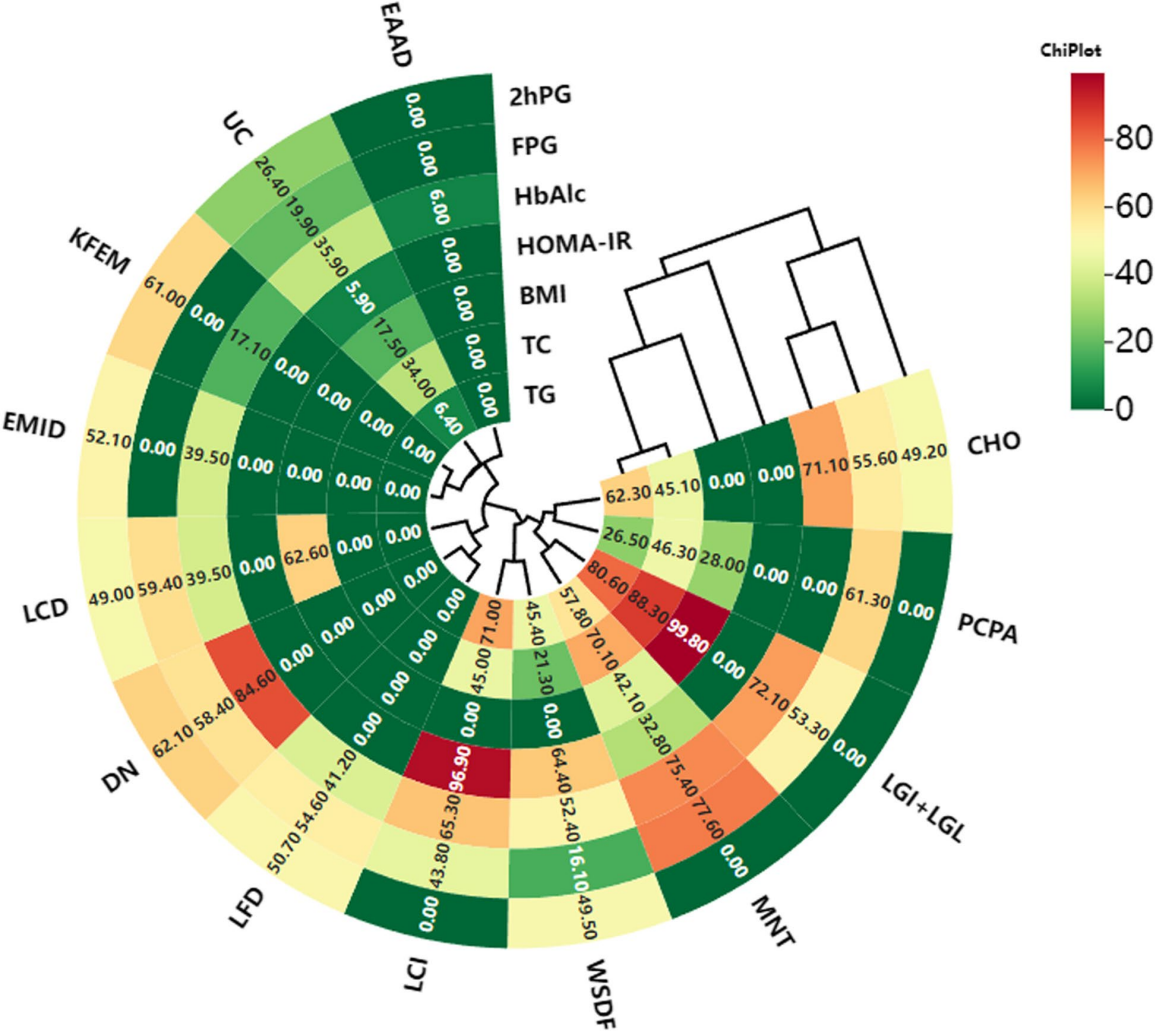
SUCRA result sort clustering heatmap

### Pairwise comparison results

Pairwise comparisons across the seven outcome indicators were conducted using league tables. For HbA1c control, the digital dietary model showed the greatest reduction compared to conventional diabetes dietary interventions (SMD = −1.06; 95% CI: −2.11 to −0.01; *P* < 0.001). MNT also demonstrated a significant advantage (SMD = −0.74; 95% CI: −1.28 to −0.19). Additionally, the Korean food exchange model (SMD = −1.68; 95% CI: −3.16 to −0.20) and East Asian alternative diet (SMD = −2.13; 95% CI: −3.64 to −0.62) were less effective than LGI + LGL (SMD = −1.79; 95% CI: −3.31 to −0.27), carbohydrate counting (SMD = −1.77; 95% CI: −3.32 to −0.22), and LGI dietary interventions (SMD = −1.61; 95% CI: −2.88 to −0.33), all showing statistically significant improvements (*P* < 0.05).

For HOMA-IR reduction, the LGI intervention was markedly more effective than both MNT (SMD = −8.04; 95% CI: −15.07 to −1.00) and conventional diets (SMD = −10.13; 95% CI: −15.96 to −4.30). The water-soluble dietary fiber intervention also significantly reduced HOMA-IR compared to conventional diets (SMD = −6.12; 95% CI: −12.14 to −0.10; *P* < 0.05). In terms of BMI reduction, the LGI + LGL intervention showed a statistically significant benefit compared to low-carbohydrate diet (SMD = −2.03; 95% CI: −3.57 to −0.49), MNT (SMD = −2.41; 95% CI: −3.88 to −0.95), PCPA (SMD = −2.66; 95% CI: −4.58 to −0.74), and conventional diets (SMD = −2.73; 95% CI: −4.03 to −1.43), with all differences being statistically significant (*P* < 0.05).

These findings underscore the relative superiority of digital dietary models, LGI-based diets, and the LGI + LGL combination in improving glycemic control, insulin resistance, and body weight among patients with T2DM. Full pairwise comparisons for all outcomes are presented in Supplementary Table X.

### SUCRA results ranking cluster heatmap

Figure 4 shows a circular quantity indicating the seven outcome indicators, and the number of sectors represents the twelve dietary interventions. The color-coded sections represent the SUCRA values of the interventions. MNT was the most effective for reducing FPG, while LGI dietary intervention was the preferred choice for reducing 2hPG and HOMA-IR. Digital dietary patterns were the most effective for reducing HbA1c, and LGI + LGL dietary interventions were the most effective for reducing TC, TG, and BMI.

### Sensitivity analyses

All seven outcome indicators formed closed-loop network structures. The global consistency test, performed using the inconsistency model, yielded *P*-values > 0.05, and the local inconsistency test based on node-splitting also showed P-values > 0.05, indicating that consistency assumptions were met across the network.

For the meta-analysis, sensitivity analyses based on heterogeneity levels demonstrated the stability of results, with minimal differences observed between the random-effects and fixed-effects models. Additionally, re-analysis after excluding low-quality studies showed no substantial impact on overall effect sizes, further supporting the robustness of the findings.

Moreover, meta-regression was conducted to explore the influence of study-level characteristics on outcome heterogeneity. The results indicated that intervention duration and study quality were the most consistent contributors to heterogeneity. Specifically, interventions lasting longer than six months were associated with greater reductions in HbA1c and BMI. In contrast, studies with higher risk of bias exhibited more variable effect estimates. These findings reinforce the validity of applying random-effects models in this context and lend additional support to the consistency and reliability of the overall conclusions.

### Publication bias

The funnel plot for the NMA showed a symmetrical distribution of data points in the upper part of the funnel, indicating few small-sample studies. This suggests that the results are stable. The scatter points were evenly distributed, and all studies were symmetrically distributed around the vertical line at X = 0, indicating that there is a low likelihood of publication bias in the current research.

## Credibility of the evidence

### GRADE evidence rating

For traditional meta-analysis, six outcome indicators were assessed: four high-quality outcomes (FPG, 2hPG, HbA1c, BMI) and two moderate-quality outcomes (TC, TG). Among these, three outcomes (FPG, 2hPG, HbA1c) were classified as"critical"and three outcomes (BMI, TC, TG) as"important."The GRADE evidence rating and reasons for upgrading or downgrading for each outcome can be found in Table 4.

**Table 4:** Summary table of GRADE evidence

Certainty assessment								№; Patients		Outcome			
Outcome Indicator	N	Study Design	Risk of Bias	Inconsistency	Indirectness	Imprecision	Other Considerations	[Intervention]	[Control]	Relative (95% CI)	Absolute (95% CI)	Certainty	Importance
FPG	10	Randomized trials	Not serious	Not serious a	Not serious	Not serious	None	494	432	-	SMD 0.75 SD lower (0.88 lower to 0.61 lower)	⨁⨁⨁⨁ High	Critical
2hPG	8	Randomized trials	Not serious	Not serious a	Not serious	Not serious	None	329	394	-	SMD 0.62 SD lower (0.76 lower to 0.47 lower)	⨁⨁⨁⨁ High	Critical
HbAlc	12	Randomized trials	Not serious	Not serious a	Not serious	Not serious	None	649	646	-	SMD 0.45 SD lower (0.45 lower to 0.33 lower)	⨁⨁⨁⨁ High	Critical
TC	8	Randomized trials	Not serious	Serious a	Not serious	Not serious	None	392	405	-	SMD 0.39 SD lower (0.54 lower to 0.25 lower)	⨁⨁⨁◯ Moderate	Important
TG	7	Randomized trials	Not serious	Serious a	Not serious	Not serious	None	330	326	-	SMD 0.59 SD lower (0.75 lower to 0.43 lower)	⨁⨁⨁◯ Moderate	Important
BMI	5	Randomized trials	Not serious	Not serious	Not serious	Not serious	None	259	244	-	SMD 0.28 SD lower (0.45 lower to 0.1 lower)	⨁⨁⨁⨁ High	Important

(a) Differences in unit conversions or variations in population/intervention may introduce heterogeneity across studies

*CI* Confidence interval, *MD* Mean difference, *OR* Odds ratio, *SMD* Standardized mean difference

#### CINeMA evidence rating

CINeMA assessed the NMA across six domains: within-study bias and between-study bias (no concern, no downgrade), and indirectness, imprecision, heterogeneity, and inconsistency (some concern, downgraded by one level). The evidence quality was rated high for FPG, 2hPG, HbA1c, and BMI, and moderate for HOMA-IR, TC, and TG. Overall, the evidence is of good quality and provides valuable recommendations.

## Discussion

This NMA incorporated 18 RCTs, encompassing 1,687 patients with T2DM. The overall methodological quality was rated as moderate to high based on the Cochrane Risk of Bias 5.4.0 tool and the Jadad scale, with excellent inter-rater reliability (Kappa = 0.897). Integrating traditional pairwise meta-analysis with Bayesian network meta-analysis generated comparative rankings of 12 dietary interventions across glycemic, lipid and anthropometric outcomes, offering a comprehensive evaluation of their relative efficacy in managing T2DM.

Several meta-analyses have evaluated dietary interventions for T2DM, yet they frequently focus on a narrow range of diets or omit emerging approaches such as digital platforms and PCPA frameworks. For example, Schwingshackl et al. compared eight dietary patterns in patients with T2DM, identifying the Mediterranean and low-glycemic-index diets as effective for glycemic control but excluding digital or model-based interventions [52]. Likewise, Peres et al. examined the impact of low-glycemic-index and low-glycemic-load diets in prediabetes and T2DM without assessing their effects alongside conventional or behaviorally structured regimens [45]. By incorporating 12 distinct interventions, including both traditional and technology-assisted strategies, this NMA evaluates their relative efficacy across seven clinically relevant outcomes. Results reaffirm the superiority of low-glycemic-index diets for improving 2-h postprandial glucose and HOMA-IR, in line with previous findings, and additionally highlight digital dietary models as the most effective for reducing HbA1c, an outcome not addressed in earlier analyses.

The SUCRA rankings provided a probabilistic hierarchy of intervention effectiveness across outcomes, which can inform dietary prioritization in clinical settings. For instance, MNT achieved the highest SUCRA score for reducing FPG (77.6%), reinforcing its role as a first-line dietary strategy. Similarly, digital dietary models ranked highest for HbA1c (84.6%), suggesting a strong potential for integration into glycemic control programs, particularly where real-time feedback can enhance adherence. The LGI intervention’s dominance in HOMA-IR (96.9%) and 2hPG (62.1%) highlights its mechanistic suitability for patients with pronounced postprandial dysregulation. Furthermore, the LGI + LGL approach ranked highest for BMI, TC, and TG, indicating its broader metabolic benefits. These rankings offer an evidence-based framework for selecting dietary interventions tailored to specific clinical priorities (e.g., glycemic control vs. lipid management).

In diabetes management, clearly distinguishing between"critical"and"important"outcome indicators allows healthcare providers to more effectively formulate and adjust treatment plans to minimize the risk of complications. In alignment with the GRADE framework, FPG, 2hPG, and HbA1c were classified as"critical"outcomes, given their established roles in the diagnosis, monitoring, and complication risk stratification of T2DM. Secondary outcomes such as TC, TG, and BMI were determined as"important"outcomes. The CINeMA evidence rating revealed that the quality of evidence for FPG, 2hPG, HbA1c, and BMI was high, while the quality for HOMA-IR, TC, and TG was moderate. Overall, the evidence was of good quality and offers valuable recommendations. The critical outcome indicators, (FPG, 2hPG, and HbA1c), directly reflect the control of diabetes and the potential risks of complications, making them essential for prioritization in treatment planning. On the other hand, the important outcome indicators (TC, TG, and BMI) contribute to a comprehensive assessment of treatment effects. Although they may not directly influence patient survival rates or the occurrence of complications as much as the critical indicators, they provide insight into the patient's overall health status and the comprehensive effects of the treatment. This information, in turn, helps healthcare providers deliver more personalized treatment recommendations for patients.

Among all dietary strategies, MNT is crucial in preventing and managing diabetes, effectively lowering FPG in T2DM patients, followed by PCPA dietary interventions. MNT reduces β-cell stress through structured dietary habits, optimizing caloric intake to maintain ideal weight. A balanced diet should meet energy needs while controlling total calories, with fat intake at 1 g/kg/day, protein at 1–1.2 g/kg/day, and carbohydrates derived from remaining caloric needs. Key recommendations include adequate hydration, limiting alcohol, quitting smoking, regular meals, portion control, and keeping salt intake under 6 g/day. Healthcare providers should adjust energy intake based on weight to maintain an optimal fat-protein-carb balance.

LGI dietary interventions have the highest likelihood (62.1%) of reducing 2hPG and the greatest likelihood (96.9%) of improving HOMA-IR, making LGI an optimal intervention for both. LGI foods have a longer gastrointestinal transit time, lower absorption rates, and slower digestion, which results in a more gradual and lower rise in blood glucose, leading to better control of 2hPG. T2DM patients should be encouraged to follow LGI dietary principles. LGI interventions help lower 2hPG and HOMA-IR through multiple mechanisms.

The digital dietary model [48] ranks first in lowering HbA1c, followed by MNT [20]. Although statistical significance is well established across interventions, the clinical relevance of the observed effect sizes warrants careful consideration. Prior evidence indicates that an HbA1c reduction of ≥ 0.5% is associated with a decreased risk of microvascular complications [51]. In this context, the estimated absolute HbA1c reductions of approximately 0.7–0.9% achieved with digital dietary interventions are likely clinically meaningful. Similarly, low-carbohydrate diets, typically restricting carbohydrate intake to 20–45% of total energy—attenuate postprandial glycemic excursions and insulin demand, thereby enhancing insulin sensitivity. Such diets have been shown to reduce HbA1c significantly within 3–6 months [18], and carbohydrate counting over 12 months has yielded reductions of approximately 0.8% [9]. Furthermore, medical nutrition therapy (MNT), when implemented by registered dietitians based on individualized assessments of metabolic status, comorbidity risk and dietary preferences, has been reported to reduce HbA1c by 0.3–2.0% [12]. Collectively, these findings demonstrate that the top-ranked interventions in this network meta-analysis not only exhibit statistical superiority but also deliver clinically meaningful improvements in glycemic control, thereby supporting their application in real-world diabetes management.

The combination of LGI + LGL nutritional intervention demonstrates optimal effects in lowering TC, TG, and BMI, with the effectiveness percentages ranked as follows: 88.3%, 80.6%, and 99.8%, respectively. This highlights that BMI is the most positively impacted parameter. To reduce HOMA-IR, guiding patients in choosing LGI foods is the top priority, with an effectiveness ratio of 96.9%. Employing scientific cooking methods and creating rational meal plans can effectively lower HOMA-IR. Meal plans should be tailored based on the patient's height, weight, and physical activity for optimal results. Improving insulin sensitivity, and thus insulin resistance and overall health, can be achieved by increasing dietary protein content and reducing the GI value [15].

The integration of MNT, LGI dietary intervention, digital dietary models, and LGI + LGL nutritional interventions forms a comprehensive and effective strategy for diabetes management. These approaches collectively aid in better controlling blood glucose levels, reducing the risk of cardiovascular diseases, and improving the quality of life for diabetic patients. In clinical practice, it is essential to actively promote and apply these dietary interventions to provide more comprehensive and personalized services for diabetes patients.

Future clinical settings should establish digital “Internet + ” diabetes nutrition intervention platforms that allow patients to access interventions via mobile apps. The platform should incorporate the best evidence provided by this study and offer customized dietary interventions based on MNT principles. Patients can upload metabolic data and food photos for CI calculation and access health education, exercise courses, peer support, nutritional follow-ups, and family connectivity. This approach would overcome the temporal and spatial limitations of traditional in-person dietary consultations and follow-ups. Additionally, a supervision and management system should be implemented to create a seamless “hospital-community-family” management model, addressing the ongoing management challenges of diabetes nutrition intervention [67]. Integrating online and offline platforms, this model enhances diabetes care efficiency, making comprehensive management a reality and easing the burden on patients through effective nutritional interventions.

## Strengths and limitations of the study

This study has several limitations. First, inclusion was restricted to Chinese- and English-language RCTs published between 2010 and 2024. Although this time frame was chosen to reflect contemporary evidence aligned with current clinical practice and recent advances in nutritional science and digital interventions, some earlier relevant studies may have been excluded. Second, blinding and allocation concealment were applied inconsistently across trials. Given the nature of dietary interventions, full blinding is often infeasible, which may introduce performance and detection biases and contribute to heterogeneity. Variations in baseline patient characteristics may also have influenced the magnitude of the intervention effects; however, inconsistent reporting and limited subgroup data precluded stratified analyses, highlighting the need for future studies to provide more granular patient-level data. Third, although most studies were rated as moderate to high quality based on Cochrane and Jadad assessments, variability in methodological rigor and reporting may still affect the reliability of some findings. Future research should adopt standardized reporting frameworks, such as CONSORT, to enhance transparency and minimize bias. Additionally, the geographical distribution of included studies was unbalanced, with the majority conducted in China. Cultural dietary patterns, healthcare infrastructure, and population characteristics may affect intervention adherence and efficacy, thereby limiting generalizability to other regions. Expanding the geographic scope in future research would improve external validity and better inform global dietary recommendations. Finally, most RCTs were of relatively short duration, which may not capture the long-term effects of dietary interventions on glycemic control, metabolic parameters, and diabetes-related complications. Given that type 2 diabetes mellitus is a chronic condition requiring sustained management, longer-term trials or longitudinal studies are necessary to confirm the durability and clinical relevance of the observed effects.

## Conclusions

For patients with T2DM, MNT is the most effective intervention for lowering FPG, while LGI intervention demonstrates the best results in reducing 2hPG. The digital dietary model shows significant effectiveness in lowering HbA1c. Furthermore, the combination of LGI + LGL dietary interventions exhibits potential advantages in reducing TC, TG, and BMI, but further high-quality and long-term studies are required to strengthen its credibility and confirm its long-term benefits.

## Supplementary Information

Supplementary Material 1.

Supplementary Material 2.

## Data Availability

No datasets were generated or analysed during the current study.

## Abbreviations

T2DM: Type 2 Diabetes Mellitus
RCT: Randomized Controlled Trial
NMA: Network Meta-Analysis
FPG: Fasting Plasma Glucose
2hPG: 2-Hour Postprandial Glucose
HbA1c: Glycated Hemoglobin A1c
HOMA-IR: Homeostasis Model Assessment of Insulin Resistance
TC: Total Cholesterol
TG: Triglycerides
BMI: Body Mass Index
MNT: Medical Nutrition Therapy
LGI: Low Glycemic Index
LGL: Low Glycemic Load
SUCRA: Surface Under the Cumulative Ranking Curve
GI: Glycemic Index
GL: Glycemic Load
GRADE: Grading of Recommendations Assessment, Development and Evaluation
CINeMA: Confidence in Network Meta-Analysis
