# Supplementary material for: Effects of 12 nutritional interventions on type 2 diabetes: a systematic review with network meta-analysis of randomized trials
Source: Nutr Metab (Lond). 2025 Aug 7;22:94. doi: 10.1186/s12986-025-00968-3 (PMC12329975; doi:10.1186/s12986-025-00968-3)
Supplement: Supplementary file 1 — Supplementary Material 1. [file 12986_2025_968_MOESM1_ESM.docx]

**Supplementary Table 1. Detailed Search Strategy for PubMed**

| **Step** | **Search Query** |
| --- | --- |
| #1 | "Diet"[MeSH] |
| #2 | "Diet, Mediterranean"[MeSH] |
| #3 | ("Diet"[Text Word] OR "Diets"[Text Word] OR "Food"[Text Word] OR "dietary"[Text Word] OR "eating pattern"[Text Word] OR "Mediterranean Diet"[Text Word] OR "medical nutrition intervention"[Text Word] OR "low glycemic index diet"[Text Word] OR "energy-restricted diet"[Text Word] OR "DASH"[Text Word] OR "carbohydrate-restricted diet"[Text Word] OR "fiber-enriched diet"[Text Word] OR "low-fat diet"[Text Word] OR "ethnic diet"[Text Word]) |
| #4 | #1 OR #2 OR #3 |
| #5 | "Diabetes Mellitus, Type 2"[MeSH] |
| #6 | ("Diabetes Mellitus, Type 2"[Title/Abstract] OR "type 2 diabetes"[Title/Abstract] OR "T2DM"[Title/Abstract] OR "non-insulin-dependent diabetes"[Title/Abstract] OR "adult-onset diabetes"[Title/Abstract] OR "maturity-onset diabetes"[Title/Abstract] OR "NIDDM"[Title/Abstract] OR "MODY"[Title/Abstract]) |
| #7 | #5 OR #6 |
| #8 | ("intervention"[Title/Abstract] OR "randomized controlled trial"[Title/Abstract] OR "randomized"[Title/Abstract] OR "RCT"[Title/Abstract] OR "placebo"[Title/Abstract]) |
| #9 | #4 AND #7 AND #8 |

**Supplementary Table 2. Summary of Core Search Terms in Chinese and English Databases**

| **Language** | **Databases** | **Core Search Terms** |
| --- | --- | --- |
| Chinese | CNKI, WanFang, VIP, SinoMed | 营养干预, 营养科学, 营养疗法, 营养政策, 糖尿病, 2型糖尿病, 随机对照试验 |
| English | Web of Science, PubMed, Medline, Cochrane CENTRAL | Nutritional intervention, nutrition therapy, nutrition policy, type 2 diabetes, randomized controlled trial, T2DM |
