# Supplementary material for: Effects of 12 nutritional interventions on type 2 diabetes: a systematic review with network meta-analysis of randomized trials
Source: Nutr Metab (Lond). 2025 Aug 7;22:94. doi: 10.1186/s12986-025-00968-3 (PMC12329975; doi:10.1186/s12986-025-00968-3)
Supplement: Supplementary file 2 — Supplementary Material 2. [file 12986_2025_968_MOESM2_ESM.docx]

**Low Carbohydrate Diet Intervention(LCD)**

**Low Fat Diet Intervention(LFD)**

**East Asian Alternative Diet Model(EAAD)**

**Korean Food Exchange Model(KFEM)**

**Medical nutrition therapy(MNT)**

**Carbohydrate Counting Method(CHO)**

**Digital Dietary Model(DN)**

**LGI Dietary Intervention(LGI)**

**Multifactorial Mediterranean Diet Intervention(EMID)**

**Soluble Dietary Fiber Intervention(WSDF)**

**LGI+LGL Dietary Intervention(LGI+LGL)**

PCPA dietary intervention(PCPA)

Conventional diabetes dietary intervention(UC)


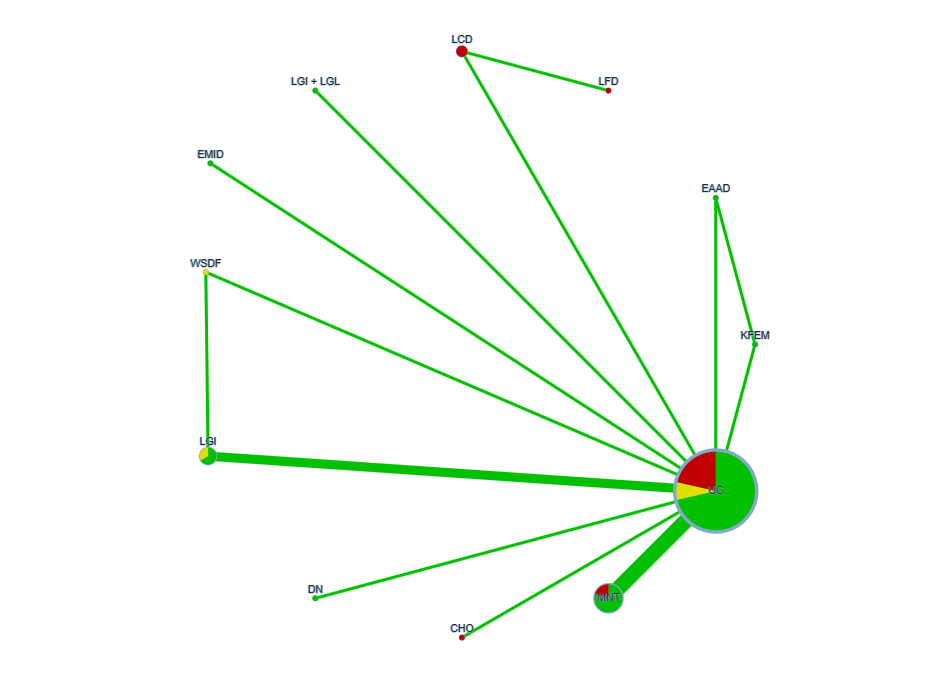


Conventional diabetes dietary intervention(UC)；**Korean Food Exchange Model(KFEM)**

**East Asian Alternative Diet Model(EAAD)；Low Carbohydrate Diet Intervention(LCD)**

**Low Fat Diet Intervention(LFD)；LGI+LGL Dietary Intervention(LGI+LGL)**

**Multifactorial Mediterranean Diet Intervention(EMID)**

**Soluble Dietary Fiber Intervention(WSDF)；LGI Dietary Intervention(LGI)**

**Digital Dietary Model(DN)；Carbohydrate Counting Method(CHO)**

**Medical nutrition therapy(MNT)**

Figure1 Network meta-analysis evidence network diagram for HbAlc


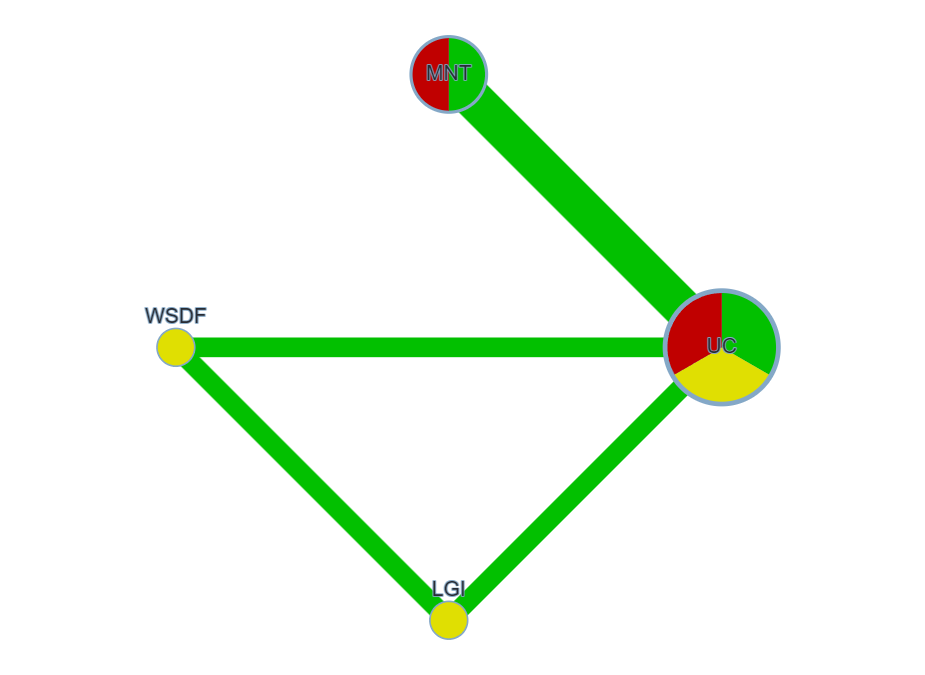


Conventional diabetes dietary intervention(UC)；**Medical nutrition therapy(MNT)**

**Soluble Dietary Fiber Intervention(WSDF)；LGI Dietary Intervention(LGI)**

Figure2 Network meta-analysis evidence network diagram for HOMA-IR


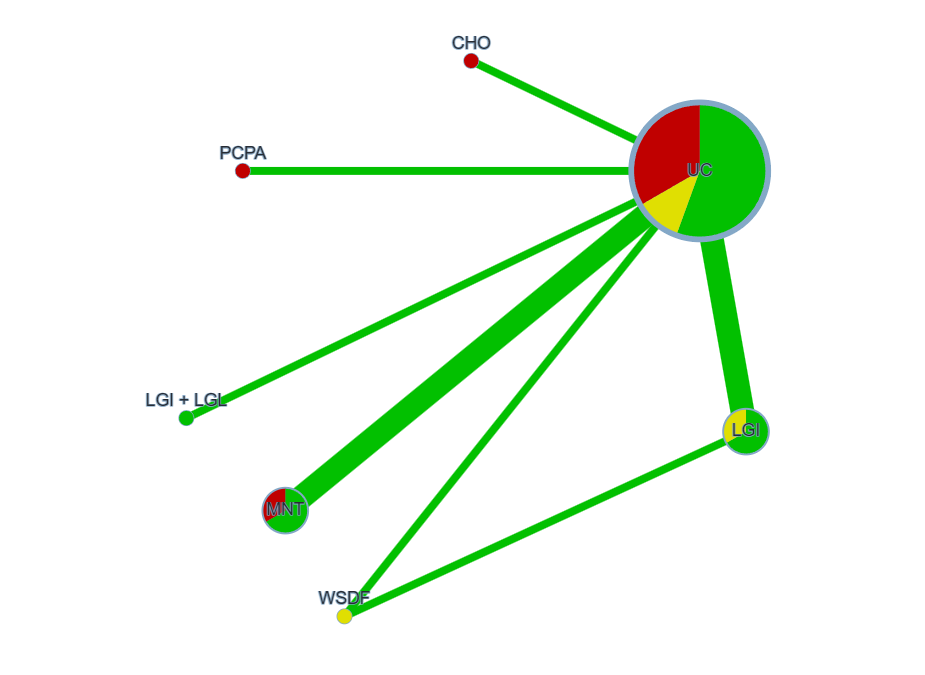


Conventional diabetes dietary intervention(UC)；**Carbohydrate Counting Method(CHO)**

PCPA dietary intervention(PCPA)；**LGI+LGL Dietary Intervention(LGI+LGL)**

**Medical nutrition therapy(MNT)；Soluble Dietary Fiber Intervention(WSDF)**

Figure3 Network meta-analysis evidence network diagram for TC


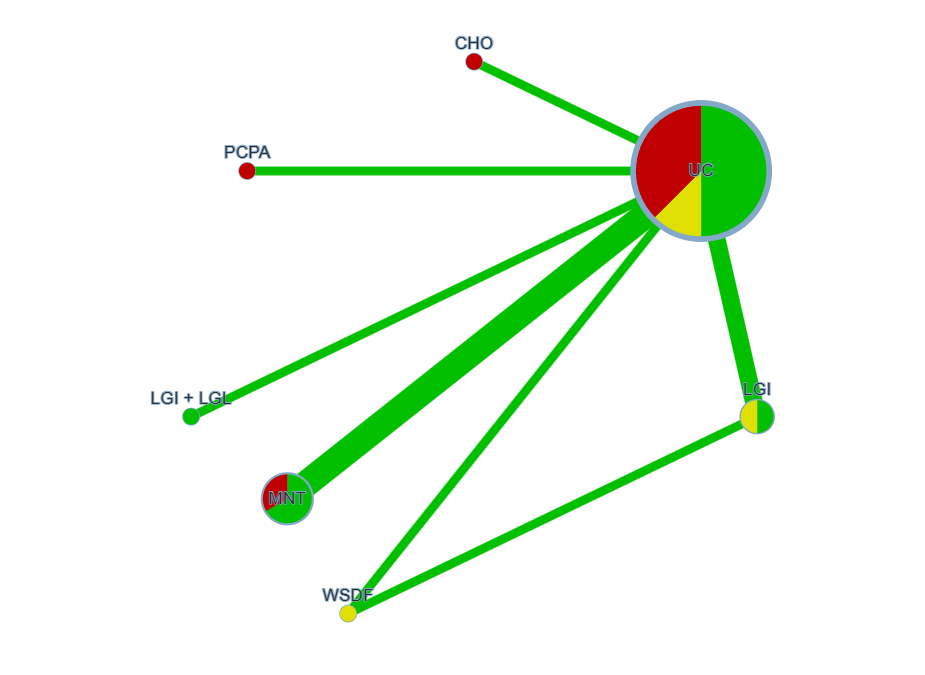


Conventional diabetes dietary intervention(UC)；**Carbohydrate Counting Method(CHO)**

PCPA dietary intervention(PCPA)；**LGI+LGL Dietary Intervention(LGI+LGL)**

**Medical nutrition therapy(MNT)；Soluble Dietary Fiber Intervention(WSDF)**

**LGI Dietary Intervention(LGI)**

Figure4 Network meta-analysis evidence network diagram for TG


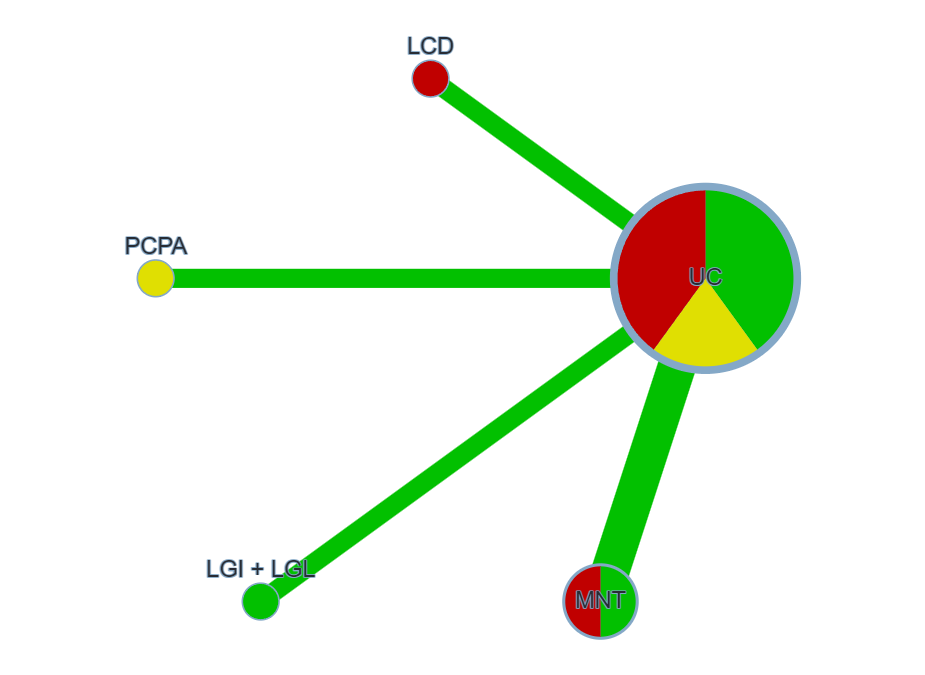


Conventional diabetes dietary intervention(UC)；**Low Carbohydrate Diet Intervention(LCD)**

PCPA dietary intervention(PCPA)**；LGI+LGL Dietary Intervention(LGI+LGL)**

**Medical nutrition therapy(MNT)**

Figure5 Network meta-analysis evidence network diagram for BMI

**Table**

**Stategy for searching literature in Pubmbed database**

| **Serial Number** | **Search Strategy** |
| --- | --- |
| **#1** | Diet[Mesh] |
| **#2** | Diet, Mediterranean[Mesh] |
| **#3** | ((((((((((((((((((Diet[Text Word]) OR (Diets[Text Word])) OR (Food[Text Word])) OR (dietary[Text Word])) OR (eating pattern[Text Word])) OR (Mediterranean Diet[Text Word])) OR (Diets, Mediterranean[Text Word])) OR (Mediterranean Diets[Text Word])) OR (Diet, Mediterranean[Text Word])) OR (Mediterranean eating pattern[Text Word])) OR (Mediterranean-style diet[Text Word])) OR (medical nutrition intervention[Text Word])) OR (Low glycemic index diet[Text Word])) OR (Energy-restricted diet[Text Word])) OR (Dietary Approaches to Stop Hypertension, DASH[Text Word])) OR (Carbohydrate-restricted diet[Text Word])) OR (Fiber-enriched diet[Text Word])) OR (Low-fat diet[Text Word])) OR (Ethnic diet[Text Word]) |
| **#4** | **#1 OR #2 OR #3** |
| **#5** | Diabetes Mellitus, Type 2[Mesh] |
| **#6** | ((((((((((((((((((((((((((((((((((Diabetes Mellitus, Type 2[Title/Abstract]) OR (Diabetes Mellitus, Noninsulin-Dependent[Title/Abstract])) OR ("type 2 diabetes[Title/Abstract])) OR (diabetes type 2[Title/Abstract])) OR (diabetic[Title/Abstract])) OR (Diabetes Mellitus, Ketosis-Resistant[Title/Abstract])) OR (Diabetes Mellitus, Ketosis Resistant[Title/Abstract])) OR (Ketosis-Resistant Diabetes Mellitus[Title/Abstract])) OR (Diabetes Mellitus, Non Insulin Dependent[Title/Abstract])) OR (Diabetes Mellitus, Non-Insulin-Dependent[Title/Abstract])) OR (Non-Insulin-Dependent Diabetes Mellitus[Title/Abstract])) OR (Diabetes Mellitus, Stable[Title/Abstract])) OR (Stable Diabetes Mellitus[Title/Abstract])) OR (Diabetes Mellitus, Type II[Title/Abstract])) OR (NIDDM[Title/Abstract])) OR (Diabetes Mellitus, Noninsulin Dependent[Title/Abstract])) OR (Diabetes Mellitus, Maturity-Onset[Title/Abstract])) OR (Diabetes Mellitus, Maturity Onset[Title/Abstract])) OR (Maturity-Onset Diabetes Mellitus[Title/Abstract])) OR (Maturity Onset Diabetes Mellitus[Title/Abstract])) OR (MODY[Title/Abstract])) OR (Diabetes Mellitus, Slow-Onset[Title/Abstract])) OR (Diabetes Mellitus, Slow Onset[Title/Abstract])) OR (Slow-Onset Diabetes Mellitus[Title/Abstract])) OR (Type 2 Diabetes Mellitus[Title/Abstract])) OR (Noninsulin-Dependent Diabetes Mellitus[Title/Abstract])) OR (Noninsulin Dependent Diabetes Mellitus[Title/Abstract])) OR (Maturity-Onset Diabetes[Title/Abstract])) OR (Diabetes, Maturity-Onset[Title/Abstract])) OR (Maturity Onset Diabetes[Title/Abstract])) OR (Type 2 Diabetes[Title/Abstract])) OR (Diabetes, Type 2[Title/Abstract])) OR (Diabetes Mellitus, Adult-Onset[Title/Abstract])) OR (Adult-Onset Diabetes Mellitus[Title/Abstract])) OR (Diabetes Mellitus, Adult Onset[Title/Abstract]) |
| **#7** | **#5 OR #6** |
| **#8** | ((((intervention[Title/Abstract]) OR (randomized controlled trial[Title/Abstract])) OR (randomized[Title/Abstract])) OR (RCT[Title/Abstract])) OR (placebo[Title/Abstract]) |
| **#9** | #4 AND #7 AND #8 |

**Results of Traditional Meta-Analysis and Heterogeneity Test (SMD, 95%CI)**

| **Outcome Indicator** | **Number of Included Studies** |  | **Sample Size** | |  | **Heterogeneity Test** | |  | **Traditional Meta-Analysis Results** | | |
| --- | --- | --- | --- | --- | --- | --- | --- | --- | --- | --- | --- |
|  |  | **Intervention Comparison** | **T** | **C** |  | ***I^2^*** | ***P*** | **Model** | **SMD** | **95%CI** | ***P*** |
| FPG | 10 | ②⑥⑦⑧⑨⑫⑬VS① | 494 | 432 |  | 88.3% | 0.000 | Random | -0.75 | (-0.88~-0.61) | 0.000 |
| 2hPG | 8 | ②⑥⑧⑨⑫VS① | 329 | 394 |  | 78.5% | 0.000 | Random | -0.62 | (-0.76~-0.47) | 0.000 |
| HbAlc | 12 | ②④⑤⑥⑦⑧⑨⑩⑫VS① | 649 | 646 |  | 80.4% | 0.000 | Random | -0.45 | (-0.45~-0.33) | 0.000 |
| TC | 8 | ⑥⑦⑨⑫⑬VS① | 392 | 405 |  | 93.5% | 0.000 | Random | -0.39 | (-0.54~-0.25) | 0.000 |
| TG | 7 | ⑥⑦⑨⑫⑬VS① | 330 | 326 |  | 82.8% | 0.000 | Random | -0.59 | (-0.75~-0.43) | 0.000 |
| BMI | 5 | ②⑥⑫⑬VS① | 259 | 244 |  | 61.8% | 0.002 | Random | -0.28 | (-0.45~-0.10) | 0.033 |
| Outcome Indicators ①conventional diabetes diet ②low-carb diet ③low-fat diet④East Asian alternative diet⑤Korean food exchange model⑥**MNT**⑦carbohydrate counting⑧digital dietary patterns ⑨LGI diet⑩multi-factor Mediterranean diet intervention ⑪water-soluble dietary fiber intervention ⑫LGI+LGL⑬PCPA dietary intervention | | | | | | | | | | | |

**HbAlc、HOMA-IR、BMI league table (MD, 95% CI)**

| Items (MD, 95% CI) | Digital Nutrition-Based Dietary Model | Personalized Medical Nutrition Therapy (MNT) | Low-Glycemic Index and Load Intervention | Carbohydrate Counting | Low-Glycemic Index (LGI) Dietary Intervention | Water-Soluble Dietary Fiber Intervention |
| --- | --- | --- | --- | --- | --- | --- |
| **1.HbAlc league table** |  |  |  |  |  |  |
| Routine Diabetes Dietary Intervention | -1.06 (-2.11,-0.01) | -0.74 (-1.28,-0.19) | - | - | - | - |
| Korean Food Exchange Model | -1.68 (-3.16,-0.20) | -1.36 (-2.52,-0.19) | - | - | - | - |
| East Asian Alternative Dietary Model | -2.13 (-3.64,-0.62) | -1.81 (-3.02,-0.60) | -1.79 (-3.31,-0.27) | -1.77 (-3.32,-0.22) | -1.61 (-2.88,-0.33) |  |
| **2.HOMA-IR league table** |  |  |  |  |  |  |
| Personalized Medical Nutrition Therapy (MNT) | - | - | - | - | -8.04 (-15.07,-1.00) |  |
| Routine Diabetes Dietary Intervention | - | - | - | - | -10.13 (-15.96,-4.30) | -6.12 (-12.14,-0.10) |
| **3.BMI league table** |  |  |  |  |  |  |
| Low-Carbohydrate Dietary Intervention | - | - | -2.03 (-3.57,-0.49) | - | - | - |
| Personalized Medical Nutrition Therapy (MNT) | - | - | -2.41 (-3.88,-0.95) | - | - | - |
| PCPA Dietary Intervention | - | - | -2.66 (-4.58,-0.74) | - | - | - |
| Routine Diabetes Dietary Intervention | - | - | -2.73 (-4.03,-1.43) | - | - | - |
